# Nanosilver Colloid Inhibits *Toxoplasma gondii* Tachyzoites and Bradyzoites in Vitro

**Published:** 2019

**Authors:** Saeedeh SHOJAEE, Nima FIROUZEH, Hussein KESHAVARZ, Sanaz JAFAR-POUR AZAMI, Mahboobeh SALIMI, Mehdi MOHEBALI

**Affiliations:** 1. Department of Medical Parasitology and Mycology, School of Public Health, Tehran University of Medical Sciences, Tehran, Iran; 2. Center for Research of Endemic Parasites of Iran (CREPI), Tehran University of Medical Sciences, Tehran, Iran

**Keywords:** Nanosilver, Electronmicroscopy, Tachyzoite, Bradyzoite, *Toxoplasma gondii*

## Abstract

**Background:**

*Toxoplasma gondii*, the coccidian protozoan parasite with worldwide distribution, is the agent of toxoplasmosis. The disease is life threatening in congenital form and in immunocompromised patients. The present study was carried out in 2016 to evaluate the in vitro effects of nanosilver colloid on tachyzoites and bradyzoites of *T. gondii*, RH and Tehran strains.

**Methods:**

Different concentrations (5, 10, 20 ppm) of nanosilver colloid were added to tachyzoites of *T. gondii*, RH strain (type I) and bradyzoites and tissue cysts of *T. gondii*, Tehran strain (type II) and incubated for 30, 60, 90 and 120 minutes. The mortality rates of tachyzoites and bradyzoites were evaluated by trypan blue dye and MTT assay. Then SEM carried out to show the changes between control and exposed parasites.

**Results:**

The greatest mortality rate was seen in 20 ppm concentration and after 120 minutes of exposure. By electron microscopy, the structural changes were seen in tachyzoites of RH and tissue cyst of Tehran strain in comparison with control groups.

**Conclusion:**

Nanosilver colloid was effective on both tachyzoites and bradyzoites of *T. gondii,* RH and Tehran strains.

## Introduction

Toxoplasmosis is an infectious disease caused by the intracellular protozoan parasite, *Toxoplasma gondii.* If a woman is infected during the pregnancy, it could be life threatening for developing fetus (1). Although recent researches show a possible relation between *T. gondii* infection and some disorders such as anxiety and cognitive disorders, schizophrenia, hyperalgesia and Alzheimer's disease in human and in experimental models, reactivated infection could be lethal in immunocompromised persons such as AIDS patients and transplant candidates (2–5).

There are three infective stages in the life cycle of *T. gondii*, the fast dividing tachyzoite, the tissue cyst containing slow growing bradyzoites and the oocyst containing sporozoites (1). The routine therapy for toxoplasmosis is the combination of sulfadizine and pyrimethamine. In addition to having serious side effects especially during the pregnancy, these drugs are effective just on tachyzoites (6). Silver, the metal with known antimicrobial, antifungal, anti-protozoan and antiviral effects could be used as an antiseptic agent in a variety of materials (7). For the antimicrobial effect, one of the most widely used nanomaterials is nanosilver which is prepared by adding Ag-No3 in stabilizers such as sodium citrate followed by adding the reducing agents such as brohidrate (8). The inhibitory effects of silver nanoparticles on *Leishmania* promastigote proliferation had been reported earlier (9). Anti-protozoan effects of this nanoparticle had been shown on various parasites such as: *Giardia lamblia*, *Entamoeba histolytica*, *Cryptosporidium parvum*, *Acanthamoeba* and *T. gondii* as well (10–13).

The present study was performed to evaluate the effects of nanosilver colloid on *T. gondii* tachyzoites of RH strain and bradyzoites and tissue cysts of Tehran strain.

## Materials and Methods

### Parasite

This study was carried out in 2016. Briefly, tachyzoites of *T. gondii*, RH strain (type I) were inoculated in peritoneal cavity of BALB/c mice as previously described (14). After 3 days of infection tachyzoites were harvested from peritoneal cavity and washed with phosphate-buffered saline (PBS, pH 7.2). Bradyzoites of *T. gondii*, Tehran strain (type II) were inoculated in peritoneal cavity of BALB/c mice. After 2 month of infection, the mice were sacrificed and their brains were examined for the presence of tissue cysts by light microscopy. By using percoll 45% (Sigma, USA) tissue cysts were isolated from the brain tissues. Some of the tissue cysts remained intact and the others were ruptured to release the bradyzoites.

Ethics Committee of Tehran University of Medical Sciences approved the study.

### Nanosilver

Commercial nanosilver colloid (Nanocid, Iran) with the size of 70 nm was used. The concentrations of 5, 10, 20 ppm from commercial nanosilver were prepared (15).

### Lethal effects of nanosilver colloid on T. gondii

Tachyzoites and bradyzoites of *T. gondii* RH and Tehran strains (1×10^4^) were exposed with these concentrations for 30, 60, 90 and 120 minutes at room temperature, respectively. At the same time tachyzoites and bradyzoites of these strains were exposed with PBS (pH 7.2) as control group. Trypan blue dye (Sigma, USA) and observing with light microscopy was performed to estimate the number of dead parasites and then, the mortality rates were calculated. The intact tissue cysts of Tehran strain were incubated with the mentioned nanosilver concentrations, dyed with trypan blue and examined with light microscopy. Experiments were carried out duplicated.

### MTT assay [3-(4, 5-dimethylthiazol-2-yl)-2, 5-diphenyltetrazolium bromide]

The MTT was performed to estimate the viability rate of tachyzoites and bradyzoites of *T. gondii* strains based on cytotoxicity assay. Briefly, after incubation of parasites with different concentrations (5, 10, 20 ppm) of nanosilver colloid, 10 μl of MTT( Zistiede, Iran) was added and incubated for 4 hours in 37 °C, then 50 μl of DMSO solution (Dimethylsulfoxid, Merck, Germany) was added. The optical density was determined at 630 nm by an automated ELISA-reader (BIOTEC, LX800, USA). The viability rate was estimated for each sample according to the formula: OD test- OD blank / OD control- OD blank ×100. Then the mortality rate was calculated. Experiments were carried out duplicated.

### Scanning electron microscopy (SEM)

For the scanning electron microscopy (SEM) the suspension of tachyzoites with the concentration of 20 ppm of nanosilver colloid was incubated for 2 hours. This procedure was done for tissue cysts as well. Samples were fixed in glutaraldehyde for 1 hour, stained with osmium tetroxide (OSO4), dehydrated, stained with gold and examined using scanning electron microscopy (Ziess, FOSUT, Germany).

## Results

**Fig. 1:**
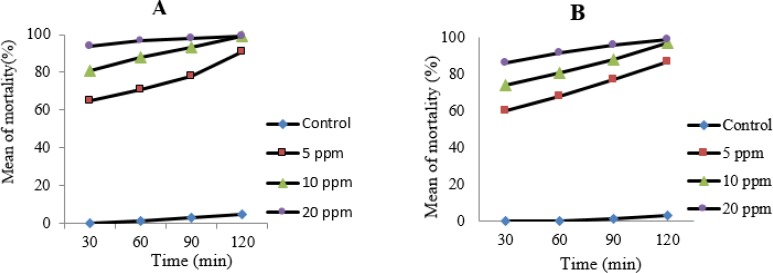
The mortality rate of tachyzoites of *T. gondii*, RH strain after 30, 60, 90 and 120 minutes of exposure with 5, 10 and 20 ppm of nanosilver colloid in comparison with control group, A) stained by trypan blue and counted by light microscopy, B) by MTT assay

Nanosilver colloid was effective in concentrations of 5, 10 and 20 ppm after 30, 60, 90 and 120 min of exposure both for tachyzoites and bradyzoites of *T. gondii*, RH and Tehran strains. The highest mortality rate was seen in 20 ppm and after 120 minutes of exposure. The results nanosilver exposed tachyzoites that stained by trypan blue and counted by light microscopy and the results of MTT assay are shown in Fig. 1 (a,b).

**Fig. 2:**
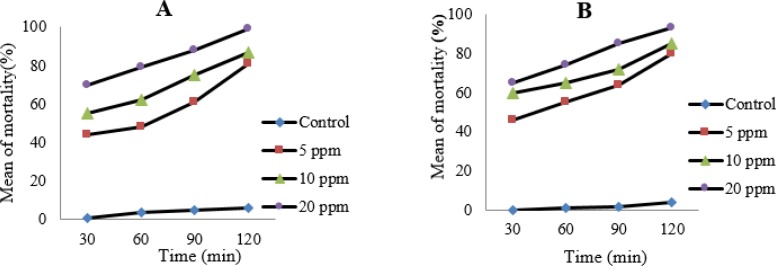
The mortality rate of bradyzoites of *T. gondii*, Tehran strain after 30, 60, 90 and 120 minutes of exposure with 5, 10 and 20 ppm of nanosilver colloid in comparison with control group, A) stained by trypan blue and counted by light microscopy, B) by MTT assay

**Fig. 3:**
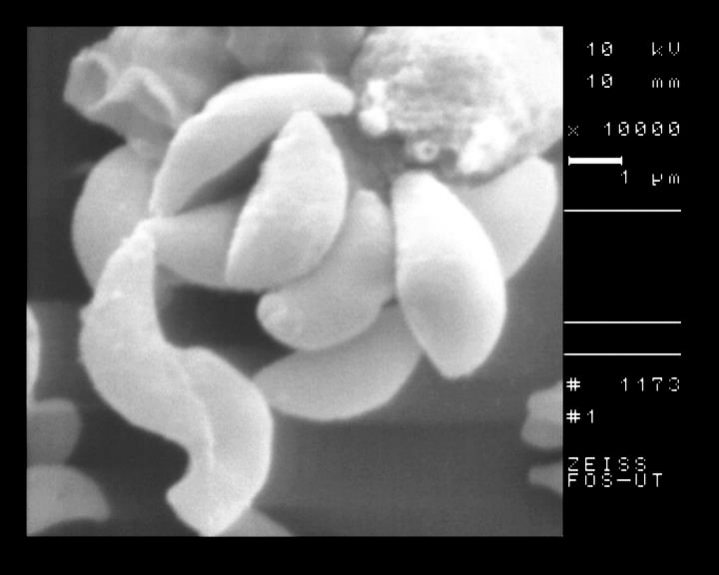
SEM of tachyzoites of *T. gondii*, RH strain in control group

**Fig. 4:**
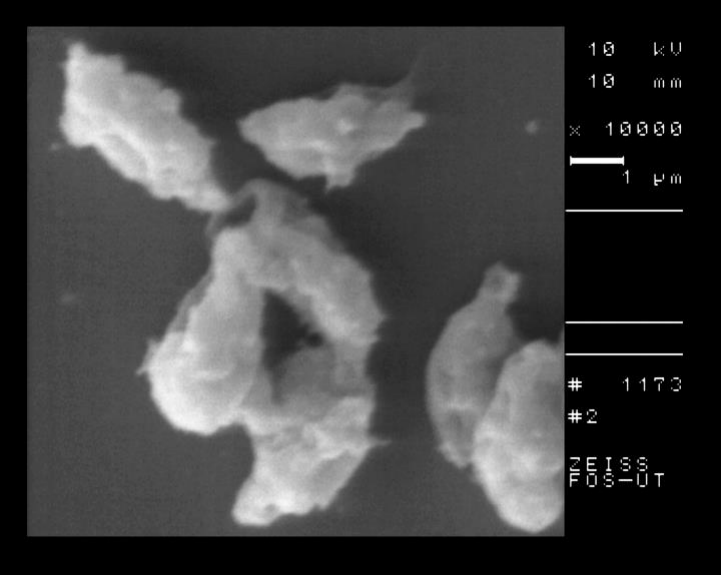
SEM of tachyzoites of *T. gondii*, RH strain after 2 hours of incubation with 20 ppm of nanosilver colloid

The results of bradyzoites of *T. gondii*, Tehran strain that exposed with the concentrations of nanosilver colloid, stained with trypan blue and counted by light microcroscopy and the results of MTT assay were shown in Fig. 2 (a,b). Results of SEM showed normal crescent shape with evident micropores in tachyzoites of control group (Fig. 3), whereas the tachyzoites that exposed with nanosilver colloid had structural changes, the crescent shape was damaged and dimples were seen in the surface of the parasite (Fig. 4).

**Fig. 5:**
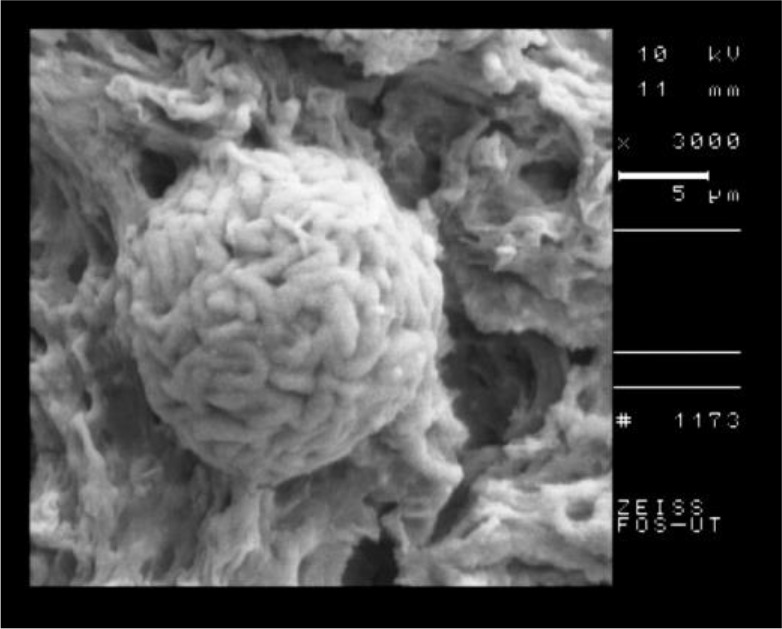
SEM of tissue cyst of *T. gondii*, Tehran strain in control group

**Fig. 6:**
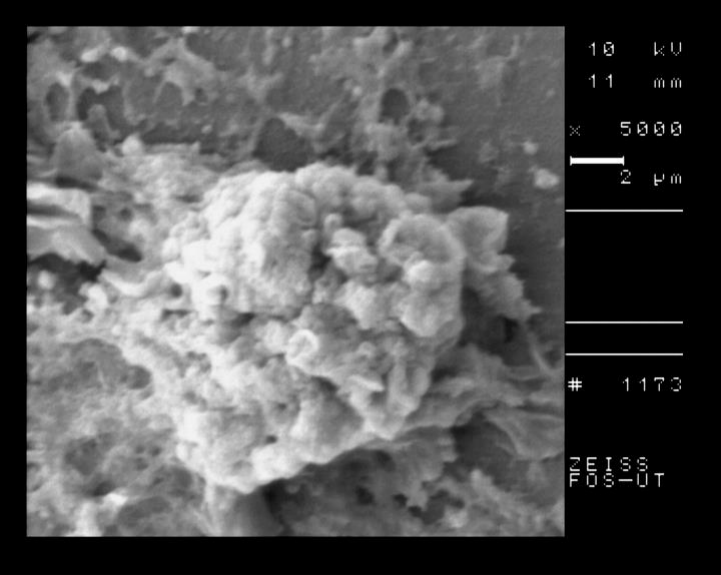
SEM of *T. gondii*, Tehran strain after 2 hours of incubation with nanosilver colloid

The SEM of the intact tissue cyst of control group is shown in Fig. 5 and the nanosilver exposed cyst is shown in Fig. 6. According to the SEM, the shape of tissue cyst in control group was normal and the bradyzoites were well organized. In contrast to the control group, the nanosilver exposed cyst was damaged with irregular surface and the bradyzoites were disorganized.

## Discussion

The standard therapy of *T. gondii* infection is usually effective on active tachyzoites form whereas treatment is ineffective for the chronic form of infection with the presence of bradyzoites (16). The biocidal activity and high temperature stability of antimicrobial polymers containing silver have attracted attentions in recent years and nanotechnology researches have focused on diagnosis and treatment of diseases by these polymers over the last decade (17–18). Cho et al. have investigated different mechanisms for killing of bacteria by nanosilver; the ability of strong binding with sulfur and phosphor compounds, damaging the bacterial cell membrane, penetration of bacteria, enzymes and DNA containing sulfur and phosphor damaging and accumulation within mitochondria and mitochondrial function impairment (19).

Nanoparticle effects have been studied in organisms such as protozoa and insect larva. Silver the known metal is widely used because of its antimicrobial properties in consumer products (8). Willcox et al. showed the activity of silver incorporated into contact lenses against *Acanthamoeba* (13). Said et al. used silver, chitosan and curcumin nanoparticles as anti- *Giardia* agents in rat experimental model (10). They investigated that the highest effect and complete cure was gained by combination of these three nano-forms in which the parasite was eradicated from stool and intestine. Saad et al. reported the anti-parasitic activity of silver and copper oxide nanoparaticles against *E. histolytica* and *C. parvum* (11). They found that by increasing the nanoparticle concentrations the mortality rate of parasites increased.

In the present study, tachyzoites of *T. gondii*, RH strain and bradyzoites of Tehran strain were exposed with the concentrations of 5,10 and 20 ppm of nanosilver colloid for 30, 60, 90 and 120 minutes. Even in 5 ppm concentration and in 30 minutes of exposure, the nanosilver colloid was effective on both tachyzoites and bradyzoites of *T. gondii*. By increasing of nanosilver concentrations and incubation times the mortality rates for both tachyzoites and bradyzoites increased, whereas the best results obtained in 20 ppm concentration and after 120 min of exposure. In this study, the intact tissue cysts of Tehran strain (type II) were incubated with nanosilver colloid too, then dyed with trypan blue and examined by light microscopy. The bradyzoites in tissue cyst were dyed gradually and after 120 min the whole organisms were dyed blue that indicates the death of bradyzoites.

The results of SEM showed the obvious structural changes in the tachyzoites of nanosilver exposed parasites in comparison with the control group. The crescent shape of tachyzoites changed to an atypical shape with large surface dimples and ridges. The SEM of tissue cyst showed the round and well organized shape in control group in comparison with a dimpled and damaged shape in nanosilver exposed one.

## Conclusion

Nanosilver colloid could kill tachyzoites and bradyzoites of *T. gondii*. Moreover this nano-particle has the ability to enter and kill the organisms inside the tissue cyst. Further studies for silver nanoparticle effects on *T. gondii* in cell culture and experimental models are suggested.
